# Evaluation of the effectiveness of two new strains of *Lactobacillus* on obesity-induced kidney diseases in BALB/c mice

**DOI:** 10.1186/s43141-022-00427-z

**Published:** 2022-10-27

**Authors:** Ahmed M. Darwish, Dalia M. Mabrouk, Hassan M. Desouky, Abd El-Nasser Khattab

**Affiliations:** 1 Cell Biology Department, Biotechnology Research Institute, National Research Centre, DokkiGiza, 12622 Egypt; 2 Animal Reproduction and Artificial Insemination Department, Veterinary Research Institute, National Research Centre, Dokki, Giza, Egypt; 3 Genetics and Cytology Department, Biotechnology Research Institute, National Research Centre, Dokki, Giza, Egypt

**Keywords:** Gene expression, Kidney disease, *Lactobacillus*, Mice, Obesity

## Abstract

**Background:**

Kidney disease (KD) is a public health problem worldwide and is an important factor in peripheral vascular disease, arrhythmias, heart failure, acute myocardial infarction, stroke, and angina. Obesity has been indicated as an effective cause of kidney diseases. So, this study aims to use two new strains of *Lactobacillus* to reduce the metabolic disorders and kidney insufficiency associated with obesity.

**Methods:**

Fifty *BALB/c* male mice were divided into five groups (control, obesity, obesity pro1, obesity pro2, and obesity mix). The bodyweight, cholesterol profile, urea, and creatinine levels in urine and serum were all measured. Histopathological analysis and expression of *Opn*, *Vim*, *Ngal*, *Kim-1*, and *αKlotho* genes for kidney tissues were performed.

**Results:**

The results indicated that body weight, cholesterol profile, urea, and creatinine levels in serum and urine had the lowest significance (*P* ˂ 0.05) in the obesity mix group and the highest significance in the obesity group. HDL had the highest significance (*P* ˂ 0.05) in the obesity mix group and the lowest significance (*P* ˂ 0.05) in the obesity group. Expression of *Opn*, *Vim*, *Ngal*, and *Kim-1* genes was the most upregulated in the obesity group compared with the other groups, and there were nonsignificant differences (*P* > 0.05) between the obesity pro1 and obesity mix groups and the control group. Expression of *αKlotho* gene was significantly reduced (*P* ˂ 0.05) in the obesity group compared with the control group.

**Conclusion:**

This study demonstrated that the combination of pro1 and pro2 strains could reduce kidney inflammation and necrosis.

## Background

Obesity has been linked to a variety of serious diseases, including obstructive sleep apnea, type 2 diabetes, fatty liver, bile ducts, cardiovascular disease, hypertension, and kidney cancer [1–3]. It also played a significant role in the progression of chronic kidney disease [4, 5]. In fact, the effect of obesity on albuminuria and blood pressure in kidney disease was reported as early as 1923 [6] when being overweight was identified as a risk reason for death, but was neglected when cardiovascular mortality was considered the main reason for death-related obesity [7]. As determined by meta-analysis, kidney disease is correlated with being overweight (*BMI* = 25–29.9) and obese (*BMI* > 30) [8]. Obesity has pathophysiological effects on the kidneys through the production of adipose tissue, inflammation, and modifications in renal hemodynamics and growth factor [9]. As a result, obesity leads to increased renal metabolic demand and expansion of the renal mesangial, resulting in hypertension, hypertrophy of the kidney, and glomerular hyper filtration, increasing glomerular filtration fraction, and subsequent glomerulosclerosis and proteinuria [10]. Moreover, obesity affects the advanced loss of kidney function among those with chronic kidney disease (CKD) [7] and causes cell carcinoma of the kidney [11].

Probiotics have multifactorial physiological functions such as controlling nutrient absorption and metabolism, regulating the normal function of the intestinal barrier, and preventing the spread of pathogenic organisms [12]. Probiotics have the ability to reduce the production of uremic toxins and to improve renal function because they have multifactorial physiological functions [13]. Chronic inflammation is considered a biomarker inversely associated with kidney function, and probiotics may control this inflammation [14]. *Fibroblast growth factor 23* (*FGF23*) is released from osteoblasts and osteocytes, and it regulates vitamin D activation 6 and renal phosphate reabsorption [15]. *FGF23* needs α-Klotho as a co-receptor for binding to fibroblast growth factor receptors (FGFRs). *α-Klotho* limits the organs targeted by *FGF23* to those where it is mainly expressed in the kidney, the parathyroid glands, and the epithelium of the choroid plexus [16]. Patients with advanced chronic kidney disease (CKD) usually display secondary hyperparathyroidism correlated with high levels of FGF23, phosphate, and a low level of 1,25(OH) 2D in serum [15]. The CKD-enhanced low level of α-Klotho expression in the parathyroid glands reduces the functionality of the α Klotho/FGFR complex for *FGF23* signalling [17]. It is also known that *FGF23* is increased from the early stage in patients with chronic kidney disease [15]. The expression of *osteopontin (Opn)*, *vimentin (Vim) neutrophil gelatinase-associated lipocalin (Ngal), and kidney injury molecule 1 (Kim-1)* genes increased significantly in the CKD model mice [13]. Although convincing epidemiological evidence suggests that excess body fat is a strong risk factor for kidney disease, the mechanisms involved in the pathogenesis of chronic kidney disease in obesity have not been fully elucidated. Therefore, this study aims to investigate the effect of obesity on kidney function through biochemical analysis and histopathological examination and to determine the expression of some genes expressed in the kidneys. In addition, evaluate the role of two new strains of probiotics in handling kidney diseases.

## Methods

### Chemicals

All the used chemicals, media, and kits were purchased from Sigma Aldrich Co., Ltd. (Saint Louis, MO, USA)**.**

### Induction of obesity

To study the effect of obesity as a risk factor in kidney injury, 500 mg of cholesterol and 100 mg of oxgall were added to 400 ml of drinking water and then provided to BALB/c mice to develop a mouse model by manipulation to model the human condition. After *BALB/c* mice were given drinking water containing a high level of cholesterol for 8 weeks, we successfully induced the obese mice [18].

### Preparation of probiotics supplement

In this study, *Lactobacillus plantarum* pro1 (MT505334.1) and *Lactobacillus rhamnosus* pro2 (MT505335.1) were used in this study. Twenty-milliliter culture tubes containing 10 ml of MRS medium were inoculated with a loopful of the tested probiotic bacterial strains and incubated at 37 °C overnight. An equal volume (10 ml) of a strain containing 2.5 × 10^6^/ml viable bacteria was centrifuged at 6000 rpm for 5 min and suspended with 400 ml of drinking water supplemented with 0.5 g cholesterol + 0.1 g oxgall. In the case of the mix group, 10 ml of each probiotic strain containing 2.5 × 10^6^/ml viable bacteria was centrifuged as above and suspended with 400 ml of drinking water supplemented with 0.5 g cholesterol + 0.1 g oxgall. This water was used as syrup to feed mice [19].

### Experimental design

The experimental procedure used in this investigation was approved by the Animal Care and Use Committee of the National Research Centre, Egypt. Fifty *BALB/c* mice weighing 21–24 g were divided into five groups and housed in cages at 22.2 °C, 50–5% humidity, and a light–dark cycle of 12 h. The animals were bought from Animal House at the National Research Centre. They were divided into five separate groups as follows: (1) control: fed on a normal diet (7% simple sugars, 3% fat, 50% polysaccharide, 15% protein (w/w), energy 3.5 kcal/g) for 8 weeks; (2) obesity: given 0.5 g cholesterol and 0.1 g oxgall in 400 ml of drinking water for 8 weeks; (3) obesity pro2: given 0.5 g cholesterol and 0.1 g oxgall and plus 10 ml of bacterial suspension (*L. rhamnosus* pro2) in 400 ml of drinking water for 8 weeks; (4) obesity pro1: given 0.5 g cholesterol and 0.1 g oxgall, and plus 10 ml of bacterial suspension (*L. plantarum* Pro1) in 400 ml of drinking water for 8 weeks; and (5) obesity mix: given 0.5 g cholesterol and 0.1 g oxgall and plus 10 ml of bacterial suspension (*L. rhamnosus* Pro2 and *L. plantarum* Pro1) in 400 ml of drinking water for 8 weeks. During this period, the individual’s weight for all mice in both groups was measured every 2 weeks.

### Biochemical analysis

Total cholesterol (TC), triglyceride (TG), low-density lipoprotein (LDL), high-density lipoprotein (HDL)-cholesterol levels, urea, and creatinine were measured in serum at 546 nm using commercial diagnostic kits by spectrophotometer model UV-240, Shimadzu (Burladingen, Germany).

### Kidney histopathology

The samples of kidney tissues were fixed in 10% neutral buffered formalin, dehydrated in alcohol, cleared in benzene, and embedded in paraffin wax. Sections of 5 μl thickness were prepared and stained with hematoxylin and eosin (H&E). Light microscopy was used in histopathological examination, and then photomicrographs were taken.

### Gene expression

The left kidneys were suspended and homogenized in TRIZOl reagent to extract total RNA according to the manufacturer’s instructions. RNA was run on an ethidium bromide-stain agarose gel to assess its integrity. The quantity and purity of RNA were measured by a NanoDrop spectrophotometer (2000c, Thermo Fisher Scientific, Wilmington, Delaware, USA). One microgram of RNA was treated with the RQ1 RNase-free DNase kit (Promega, Madison, WI, USA) to get rid of any gDNA contamination. DNase-treated RNA was transcribed into cDNA using the COSMO cDNA synthesis kit (willowfort.co.uk) according to the manufacturer’s instructions. Primer sequences were synthesized by Macrogen Co., Ltd., Korea (Table 1). Real-time quantitative PCR (RT-qPCR) analysis was carried out on the Stratagene Mx3000P Real-Time PCR System (Agilent Technologies) in a 20-μl reaction volume containing 1 μl cDNA, 0.5 μl of forward primer (10 μM) and 0.5 μl of reverse primer (10 μm), 10 μl of Hot FIREPol EvaGreen qPCR Mix Plus (Solis Biodyne, Tartu, Estonia), and 8 μl of DNAse-free water. Amplification began with a 10-min period at 95 °C, followed by 40 cycles of 30 s at 95 °C, 30 s at 60 °C, and 30 s at 72 °C. The relative expression levels of genes normalized to β-actin were calculated using the 2−ΔΔCT method [20].

**Table 1:** Sequence of employed primers

Gene	Accession no	Nucleotide sequence 5′–3′	Size (bp)
Opn	NM_001204203.1	F5-TCCAAAGAGAGCCAGGAGAG-3′ R5-GGCTTTGGAACTTGCTTGAC-3′	66
Vim	NM_011701.4	F5-CTGCACGATGAAGAGATCCA-3′ R5-AGCCACGCTTTCATACTGCT-3	132
Ngal	NM_008491.1	F5-GAAATATGCACAGGTATCCTC-3′ R5-GTAATTTTGAAGTATTGCTTGTTT-3′	124
Kim-1	NM_001166632.1	F5-CTGGAATGGCACTGTGACATCC-3′ R5-GCAGATGCCAACATAGAAGCCC-3′	112
αKlotho	NM_013823.2	F5-CCCGATGTATGTGACAGCCAATGG-3′ R5-CTTGGGAGCTGAGCGATCACTAAG-3	175
β-actin	NM_007393.5	F5**′**-GGCACCACACCTTCTACAATG-3**′** R5**′**-GGGGTGTTGAAGGTCTCAAAC-3**′**	74

### Statistical analysis

A one-way ANOVA test was performed on the observed data using SPSS (PASW statistics software version 18). The significant differences between the means were calculated using Duncan at *P* < 0.05.

## Results

### Body weight

Bodyweight measurements showed a significant increase in the obesity group (*P* ˂ 0.05) compared with the other groups and a significant reduction (*P* ˂ 0.05) in the obesity pro1 group compared with the other groups. There were nonsignificant differences (*P* > 0.05) between the obesity mix group and the control group (Table 2).

**Table 2:** Body weight of mice during the experiment period in all groups

	Control	Obesity	Obesity pro2	Obesity pro1	Obesity mix
Zero time	22 ± 0.6	22.5 ± 0.5	23 ± 0.8	22.8 ± 0.7	22.6 ± 0.5
2 weeks	24.6^b^ ± 0.4	27.5^a^ ± 0.1	26.6^a^ ± 0.9	20.4^c^ ± 0.2	25^b^ ± 0.1
4 weeks	26.4^b^ ± 0.8	29.5^a^ ± 0.3	26.4^b^ ± 0.9	21.4^c^ ± 1.1	26^b^ ± 0.2
6 weeks	27.6^b^ ± 0.6	29.5^a^ ± 0.1	27.8^b^ ± 0.1	23.6^c^ ± 0.8	27^b^ ± 0.3
8 weeks	26.8^b^ ± 0.2	31.5^a^ ± 0.4	29.8^a^ ± 0.3	25.6^c^ ± 0.1	27.5^b^ ± 0.5

All data are expressed as the mean ± SE values within a column with different superscripts

^a,b,c^Differ significantly at *P* < 0.05

### Biochemical analysis

#### Lipid profile

The obesity group recorded the significantly highest values (P ˂ 0.05) of cholesterol, triglycerides, and LDL in serum and recorded the significantly lowest value (P ˂ 0.05) of HDL in serum compared with the other groups. The obesity mix group recorded the significantly highest value (P ˂ 0.05) of HDL in serum and recorded the significantly lowest values (P ˂ 0.05) of cholesterol, triglyceride, and LDL in serum compared with the other treated groups. There are nonsignificant differences (P > 0.05) in the cholesterol profile between the obesity pro1 group and the obesity mix group (Table 3).

**Table 3:** Lipid profile analysis

Analysis type (mg/dl)	Control	Obesitys	Obesity pro2	Obesity pro1	Obesity mix
Cholesterol	126^d^ ± 0.3	250.9^a^ ± 0.7	185.4^b^ ± 0.6	165^c^ ± 0.1	162^c^ ± 0.3
Triglyceride	102^d^ ± 0.2	220.5^a^ ± 0.1	141^b^ ± 0.9	130^c^ ± 0.2	129^c^ ± 0.1
HDL	60^c^ ± 0.8	55^d^ ± 0.3	70^b^ ± 0.4	73.6^a^ ± 1.1	74^a^ ± 0.2
LDL	48^d^ ± 0.6	139.8^a^ ± 0.1	85.2^b^ ± 0.1	65^c^ ± 0.8	63^c^ ± 0.3
Urea	52.1^b^ ± 0.4	67.3^a^ ± 0.2	47.2^c^ ± 0.3	30^d^ ± 0.3	24.9^e^ ± 0.5
Creatinine in serum	3.2^b^ ± 0.3	4^a^ ± 0.03	4^a^ ± 0.2	3.4^b^ ± 0.1	3.1^b^ ± 0.4
Creatinine in urine	158.3^c^ ± 0.2	201.3^a^ ± 0.3	200^a^ ± 0.5	175^b^ ± 0.2	155^d^ ± 0.4

*HDL* High-density lipoprotein, *LDL* Low-density lipoprotein

All data were expressed as the

mean ± SE values within a column with different superscripts

^a,b,c^Differ significantly at *P* < 0.05

#### Kidney functions

Serum content of urea recorded the highest significant value (*P* ˂ 0.05) in the obesity group compared with the other groups. There were nonsignificant differences (*P* > 0.05) observed between the obesity group and the obesity pro2 group in the level of creatinine in serum and urine. While the obesity mix group recorded the significantly lowest values (*P* ˂ 0.05) of urea levels in serum and creatinine level in urine compared with the other groups (Table 3).

#### Histopathological results

**Fig. 1:**
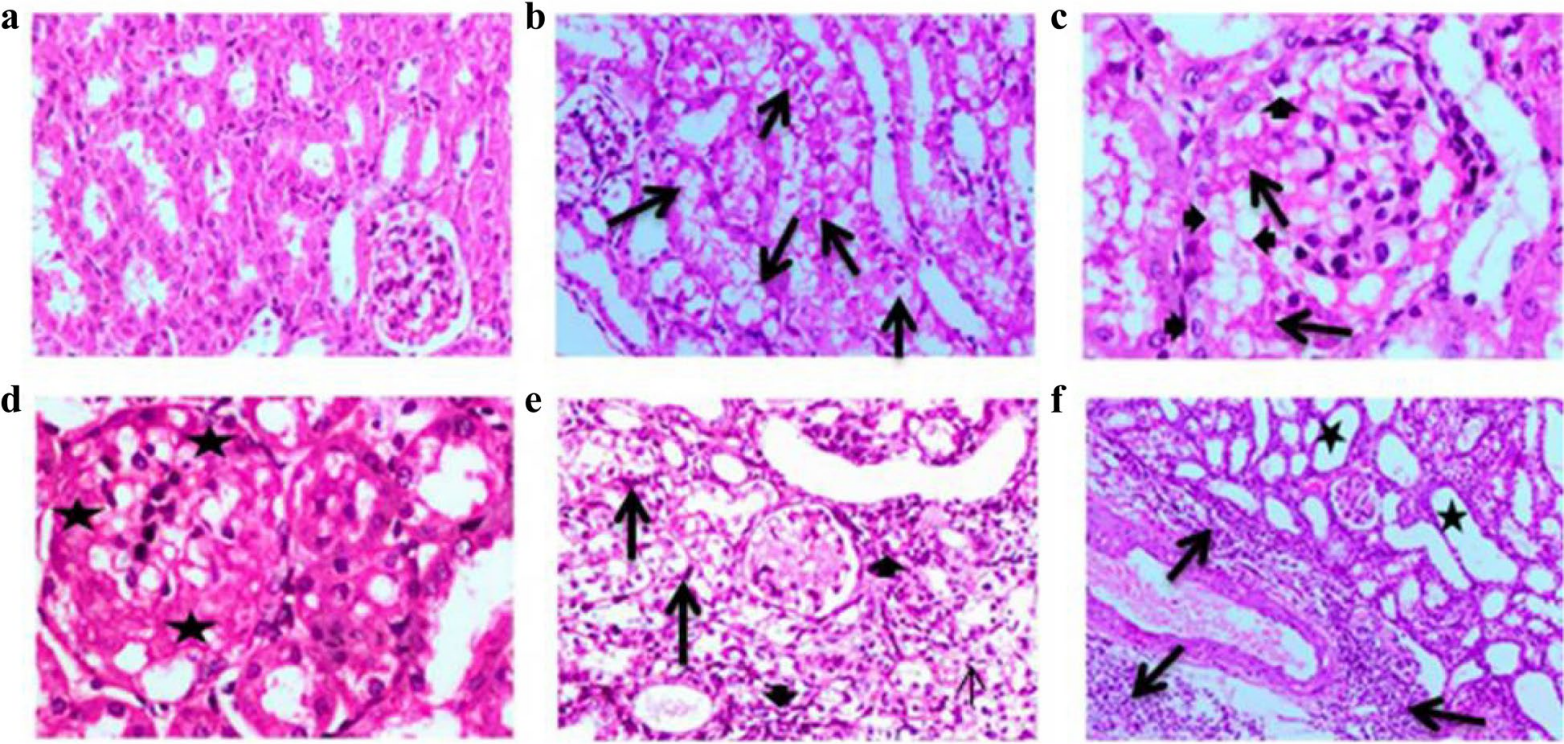
**a** Kidney of normal control group, showing normal renal tubules and glomeruli; **b** kidney of obesity group showing vacuolar degenertion of epithelial lining of most of renal tubules (black long arrows); **c** kidney of obesity group showing dilatation of glomerular capillaries with partial hyalinization of glomerulus tuft (focal glomerular sclerosis) (black long arrow) and accumulation of glomerular foam cells (black short arrows); **d** kidney of obesity group showing severe glomerular hyalinization (glomerular sclerosis) (black stars) and vacuolation of glomerular tuft; **e** kidney of obesity group showing glomerular hyalinization (glomerular sclerosis), severe necrosis of renal tubules (black long arrows), cyctic dilatation of renal tubules, and interstitial fibrosis associated with inflammatoy cell infiltration (black short arrows); and **f** kidney of obesity group, showing marked cytic dillatation of renal tubules (black stars), massive perivscular agreggations of inflammatory cells (black long arrows), dilatation, and congestion of blood vessel (H&E, × 100)

Histopathological examination of the kidney of the obesity group revealed vacuolar degeneration of the epithelial lining of most of the renal tubules (Fig. 1b). The renal glomeruli showed partial hyalinization of the glomerulus tuft (focal glomerular sclerosis) with dilatation of glomerular capillaries and accumulation of glomerular foam cells (Fig. 1c). Meanwhile, others showed severe hyalinization of the glomerular tuft (glomerular sclerosis) (Fig. 1d). In one case, renal interstitial tissue was highly infiltrated with mononuclear inflammatory cells associated with fibrosis. Moreover, degeneration and necrosis of the epithelial lining of renal tubules were noticed. Some renal tubules appeared cystically dilated and lined with flattened epithelium. Glomerular hyalinization (glomerular sclerosis) was also seen. The renal blood vessels appeared dilated and congested (Fig. 1e). Moreover, periglomerular and perivascular aggregations of mononuclear cells, mainly lymphocytes and macrophages, were observed (Fig. 1f).

**Fig. 2:**
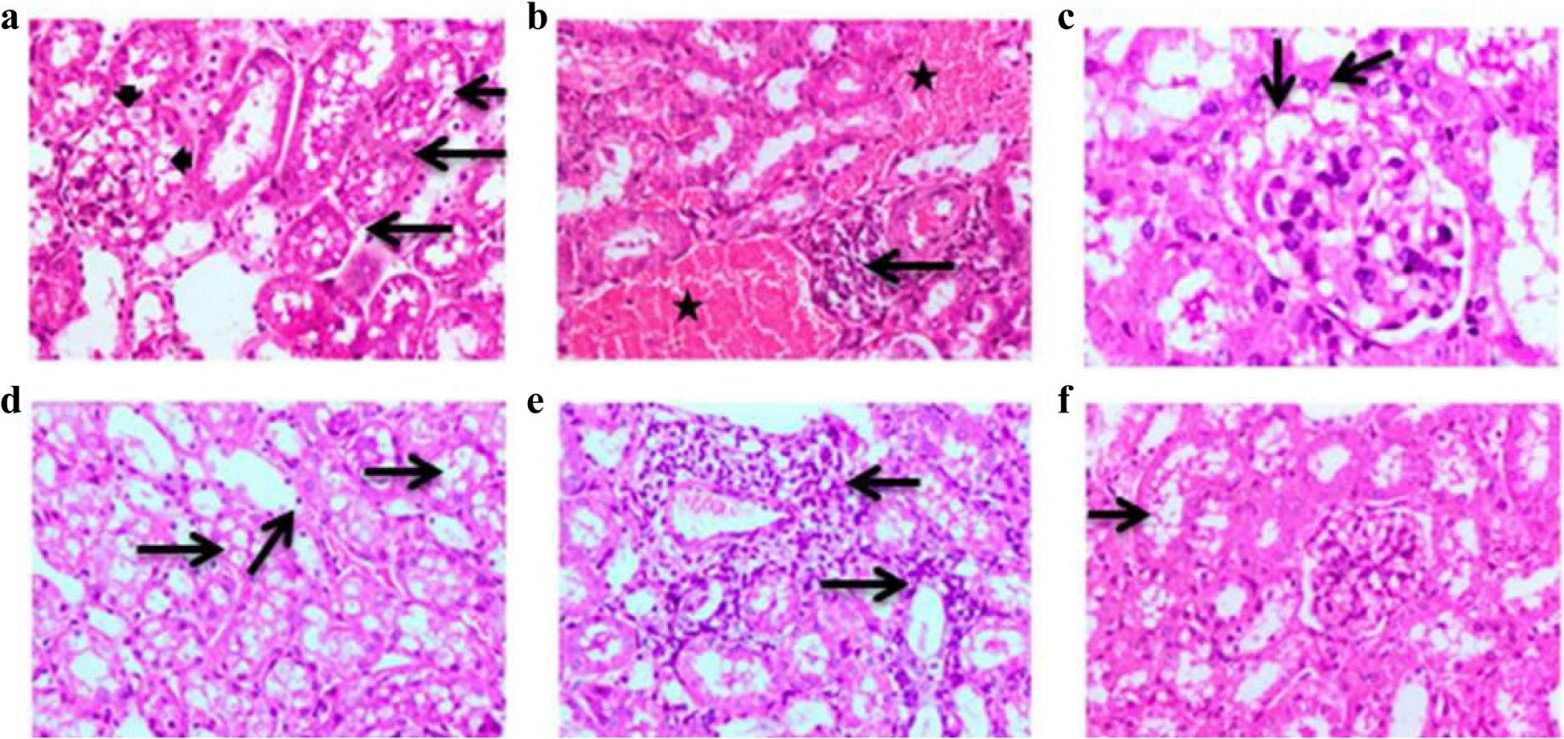
**a** kidney of obesity pro2 group, showing vacuolar degeneration and necrosis of epithelial lining of renal tubules (black long arrows), vacuolation of glomerular tuft with presence of foam cells (black short arrows), **b** kidney of obesity pro2 group, showing extensive hemorrhage, dilatation & congestion of blood vessel (black stars) and focal aggregation of inflammatory cells (black long arrows), **c** kidney of obesity pro1 group, showing marked vacuolation of glomerular tuft (black long arrows), **d** kidney of obesity pro1 group, showing vacuolar degeneration associated with necrosis of epithelial cells (black long arrows), **e** kidney of obesity mix group, showing perivascular and interstitial aggregations of mononuclear inflammatory cells (black long arrows), and (**f**) kidney of obesity mix group, showing mild vacuolar degeneration of epithelial lining of some renal tubules (black long arrows) (H&E, × 200)

The kidney of the obesity pro2 group revealed vacuolar degeneration of the epithelial lining of the renal tubules. The renal glomeruli showed vacuolation of the glomerular tuft with an accumulation of foam cells (Fig. 2a). Multifocal aggregations of inflammatory cells in the renal parenchyma associated with dilatation and congestion of renal blood vessels were observed. Moreover, there were severe medullary hemorrhages at the corticomedullary junction (Fig. 2b). The kidneys of the obesity pro1 group showed enlarged renal glomeruli associated with dilatation of glomerular capillaries and the presence of foam cells (Fig. 2c). The epithelium of renal tubules showed vacuolar degeneration associated with necrosis of epithelial cells (Fig. 2d). Focal interstitial and perivascular aggregations of mononuclear inflammatory cells were observed (Fig. 2e). The kidney of the obesity mix group revealed mild to moderate vacuolar degeneration of the epithelial lining of the renal tubules in a focal manner. The renal glomeruli appeared enlarged in size with vacuolation of glomerular capillaries (Fig. 2f). No evidence of an inflammatory reaction was seen.

#### Gene expression

**Fig. 3:**
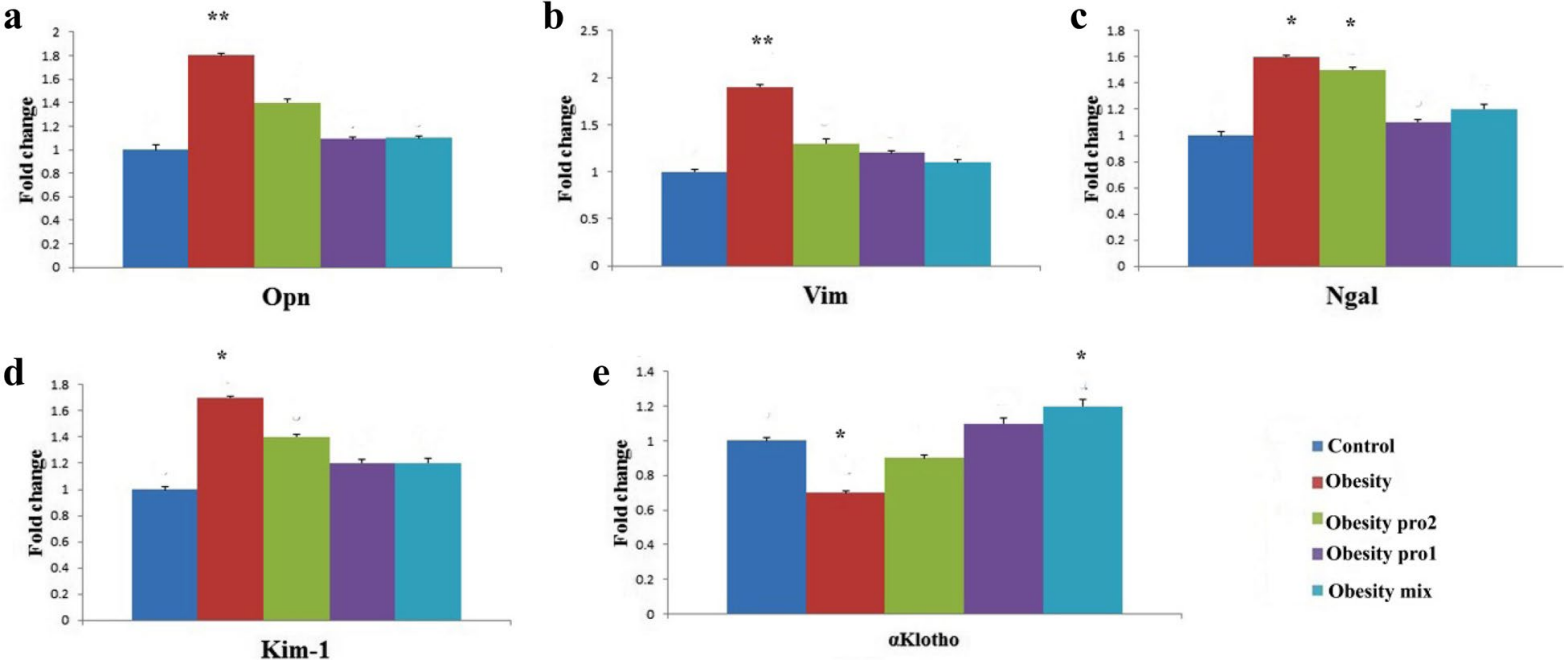
**a** shows significant up-regulation of Opn gene in the obesity group, **b** shows significant up-regulation of Vim gene in the obesity group and obesity pro2 group, **c** shows significant up-regulation of Ngal gene in the obesity group, **d** shows significant up-regulation of Kim-1 gene in the obesity group, and (**e**) shows significant down-regulation of α-Klotho gene in the obesity group and also significant up-regulation of α-Klotho gene in the obesity mix group compared with the control group

Opn, Vim, Ngal, and Kim-1 genes were significantly upregulated in the obesity group compared with the other groups. Opn, Ngal, and Kim-1 were significantly upregulated in the obesity pro2 group compared with the control. There were nonsignificant differences between the control group and the obesity mix group in the expression of Opn, Vim, Ngal, and Kim-1 genes. Expression of α-koloth was significantly increased in the obesity mix group compared with the control group while significantly downregulated in the obesity group (Fig. 3).

## Discussion

Adipose tissue acts as an endocrine and exocrine organ with neurohumoral and vasoactive effects that are associated with the development of obesity-related organ damage involving the kidney. Besides angiotensinogen and renin, adipose tissue produces growth factors, bioactive adipokines, and several cytokines correlated with kidney injury [21]. Obesity is associated with an increased risk of renal cell carcinoma [11].

Obesity causes pathophysiological changes accompanied by alterations in the inflammatory milieu, renal hemodynamics, and production of adipokine and growth factor [22]. Also, it causes renal mesangial hypertrophy and an increase in metabolic demand, as well as an increase in the glomerular filtration fraction, glomerulosclerosis, and proteinuria [10].

In the present study, obesity caused vacuolar degeneration of the epithelial lining of most renal tubules, partial hyalinization of renal glomerulus tuft with dilatation of glomerular capillaries, accumulation of glomerular foam cells, and degeneration and necrosis of the epithelial lining of renal tubules. In addition, the renal blood vessels appeared dilated and congested. Also, in the presence of periglomerular and perivascular aggregations of mononuclear cells, mainly lymphocytes and macrophages, these pathophysiological changes were associated with disorders in kidney function, such as increasing serum levels of urea and creatinine and urine levels of creatinine that significantly increased in the obesity group compared with the control group. Lower urine pH [23] and increased urinary oxalate [24], uric acid, salt, and phosphate excretion are linked to increased body weight [25]. Protein- and sodium-rich diets may cause more acidic urine and a decrease in urinary citrate, which might increase the risk of kidney stones. Obesity-related insulin resistance may further predispose to nephrolithiasis [26] by influencing the tubular Na–H exchanger [27] and ammonia genesis [28], as well as promoting an acidic environment [29]. Andrade-Oliveira et al. [30] were able to recently increase plasma short-chain fatty acids and protect mice from renal ischemia–reperfusion injury by modulating inflammation with probiotics in a mouse model of acute kidney injury. Serum levels of tumor necrosis factor- and interleukin-6, both pro-inflammatory cytokines, were reduced after 6 months of taking a probiotic capsule daily [31]. In the present study, the combination of two new strains of *Lactobacillus* reduced vacuolar degeneration of the epithelial lining of renal tubules in a focal manner. In addition, there was no observed evidence of an inflammatory reaction in the kidneys of obese mice treated with this combination. Probiotics may be able to improve renal function during treatment due to their beneficial effects, such as reducing inflammation and uremic toxins [32]. The production of t10, c12-conjugated linoleic acid by the probiotic, reduced serum leptin and fatty acid formation and thus bodyweight [33]. Also, *L. plantarum* PL62 reduced epididymal, inguinal, mesenteric, and perineal white adipose tissue mass [1935]. *L. rahmanosus* LGG increased the expression of fatty acid oxidative genes in the liver while it decreased gluconeogenic genes [34]. *L. plantarum* LG42 caused a significant reduction in epididymal and back fat and a decrease in hepatic triglyceride levels [35]. Furthermore, *L. plantarum* TN8 was found to be protective against hepatic lipid and renal profiles in obese rats [36]. These beneficial effects of probiotics accompanied by reductions in lipid accumulation may be due to stimulating adiponectin secretion and downstream activation of AMPK, an enzyme involved in controlling the energy status of cells [32]. *Blood* urea nitrogen and ammonia levels were reduced by *Bifidobacterium* genera [37]. *Lactobacillus delbrueckii* and *Sporosarcina pasteurii* hydrolyze urea in vitro and have been shown to be potential urea-targeted agents for enteric dialysis [6]. Living transgenic cells encapsulated with urease-producing *Escherichia coli* were able to reduce blood urea levels in uremic mice and reduce the conversion of urea to ammonium by living transgenic cells [38]. *Bacillus pasteurii* or *Sporosarcina pasteurii* reduced the development of kidney disease and helped to extend the life span. The probiotic LC40 improved renal function in NZBWF1 mice by increasing urinary creatinine and urea excretion and delaying the onset of albuminuria and high blood pressure [39]. In our previous study on this combination of *L. plantarum* pro1 and *L. rhamnosus pro2*, we were able to reduce diabetes-associated disorders induced by a high level of fructose [3]. In the current study, the results indicated that the level of urea in serum and the level of creatinine in urine and serum were significantly reduced in obese mice treated with the combination of new *Lactobacillus* strains compared with the other groups. In the previous study, Opn, Vim, Ngal, and Kim-1 genes were upregulated in the CKD model mice [13]. The − Klotho gene was downregulated in the parathyroid glands of patients with CKD [40]. It has been shown to protect tissues from harm by reducing oxidative stress and reducing inflammation. Moreover, − α-klotho has been demonstrated to have a protective effect on the heart and kidneys [41]. In the present study, upregulation of Opn, Vim, Ngal, and Kim-1 genes was associated with the obesity group. There is no significant difference between obese mice treated with the combination of two new *L. rhamnosus* or *L. plantarum* and control in the expression of these genes. Also, the downregulation of the α-Klotho gene was correlated with the obesity group, and it became upregulated in mice treated with a combination of two strains. Therefore, this study demonstrated that the combination of pro1 and pro2 strains could reduce kidney injury induced by obesity.

## Conclusion

Obesity increased serum creatinine and urea levels, as well as inflammation and necrosis of renal tissue and inflammation and necrosis in kidney tissues. These physiological and histopathological changes were accompanied by alterations in the genes expressed in the kidneys, such as *Opn*, *Vim*, *Ngal*, *Kim-1*, and *α-klotho* genes. The combination of two new *Lactobacillus* strains (Pro1 + Pro2) significantly reduced levels of urea and creatinine in serum and reduced inflammation in kidney tissues. Also, increased expression of *α-klotho* gene is associated with a reduction in inflammation. Therefore, using the combination of two new *Lactobacillus* strains (Pro1 + Pro2) in supplementation of many drinks such as milk or juice may be useful in reducing obesity risks associated with kidney injury.

## Data Availability

The datasets used and/or analyzed during the current study are available from the corresponding author on reasonable request.

## Abbreviations

KD: Kidney disease
TC: Total cholesterol
TG: Triglyceride
LDL: Low-density lipoprotein
HDL: High-density lipoprotein
